# Antibacterial hydrogels for skin infected wounds: frontier approaches as antibiotic alternatives therapy

**DOI:** 10.3389/fcimb.2026.1769669

**Published:** 2026-02-27

**Authors:** Xiangyang Li, Yueying Fan, Jinfu Li, Chao Yan, Peng Wang, Chiyu Jia

**Affiliations:** 1 Center for Burn, Plastic and Wound Repair Surgery, The First Affiliated Hospital of University of South China, Hengyang, China; 2 Hengyang Medical School, University of South China, Hengyang, China

**Keywords:** antibacterial hydrogels, antibacterial materials, antibiotic alternatives, skin infected wounds, stimuli-responsive, tissue regeneration, wound healing

## Abstract

Skin wound infections are common and clinically challenging. Conventional antibiotic therapies are increasingly ineffective because of escalating bacterial resistance, highlighting the urgent need for alternative treatment strategies. Antibacterial hydrogels, multifunctional polymeric materials that integrate moisturizing, drug delivery, controlled release, and wound-healing properties, have emerged as highly promising candidates for managing infected wounds. Based on their underlying antimicrobial mechanisms, these systems can be broadly classified into three main categories: chemical, physical, and biological antibacterial hydrogels, which achieve bactericidal efficacy through drug release, physical disruption, or modulation of the host microenvironment and immune responses, respectively. Of tremendous significance is the advent of stimuli-responsive intelligent hydrogels, which provides new opportunities for achieving precise and efficient antibacterial therapy. This review systematically summarizes the material selection, design strategies, and representative advances in antibacterial hydrogels, with particular emphasis on their core mechanisms, strengths, and limitations, aiming to offer theoretical foundations and research perspectives for the rational optimization and clinical translation of next-generation antibacterial hydrogels.

## 1 Introduction

The skin, as the largest organ of the human body, serves as the primary barrier against external pathogenic invasion. However, various factors, including acute and chronic trauma, diabetic foot ulcers, and postoperative wounds, frequently compromise skin barrier integrity, subsequently predisposing patients to bacterial colonization and the development of infected wounds. Clinically, the predominantly encountered wound pathogens include *Staphylococcus aureus*, *Pseudomonas aeruginosa*, and *Escherichia coli*. (Fijan et al., 2022; McLoone et al., 2021) Bacterial infections not only impede normal wound healing but can also be life-threatening by leading to sepsis and even multiple organ dysfunction syndrome in severe cases, particularly in immunocompromised patients or those with underlying comorbidities, in whom such infections occur more frequently and respond poorly to treatment (Uberoi et al., 2024).

Nevertheless, current treatment strategies remain primarily reliant on antibiotic administration, whose widespread overuse has precipitated an alarming escalation in antimicrobial resistance. (2022) Clinical investigations have shown that the proportion of infections caused by resistant strains can be as high as 60.8% among bacterial infectious disease cases. (Langford et al., 2023) One statistical estimate indicates that, without immediate intervention, global mortality attributable to antimicrobial-resistant pathogens is projected to reach approximately 10 million deaths annually by 2050 (Mancuso et al., 2021). The rapid proliferation of bacteria and complex biofilm formation render traditional treatment approaches susceptible to poor therapeutic efficacy, prolonged treatment duration, and elevated recurrence rates. (Gonçalves et al., 2023) In particular, the emergence of multidrug-resistant bacteria has further exacerbated the difficulty of infection control, making it imperative to identify safe and highly efficacious alternative treatment options.

**Figure 1:**
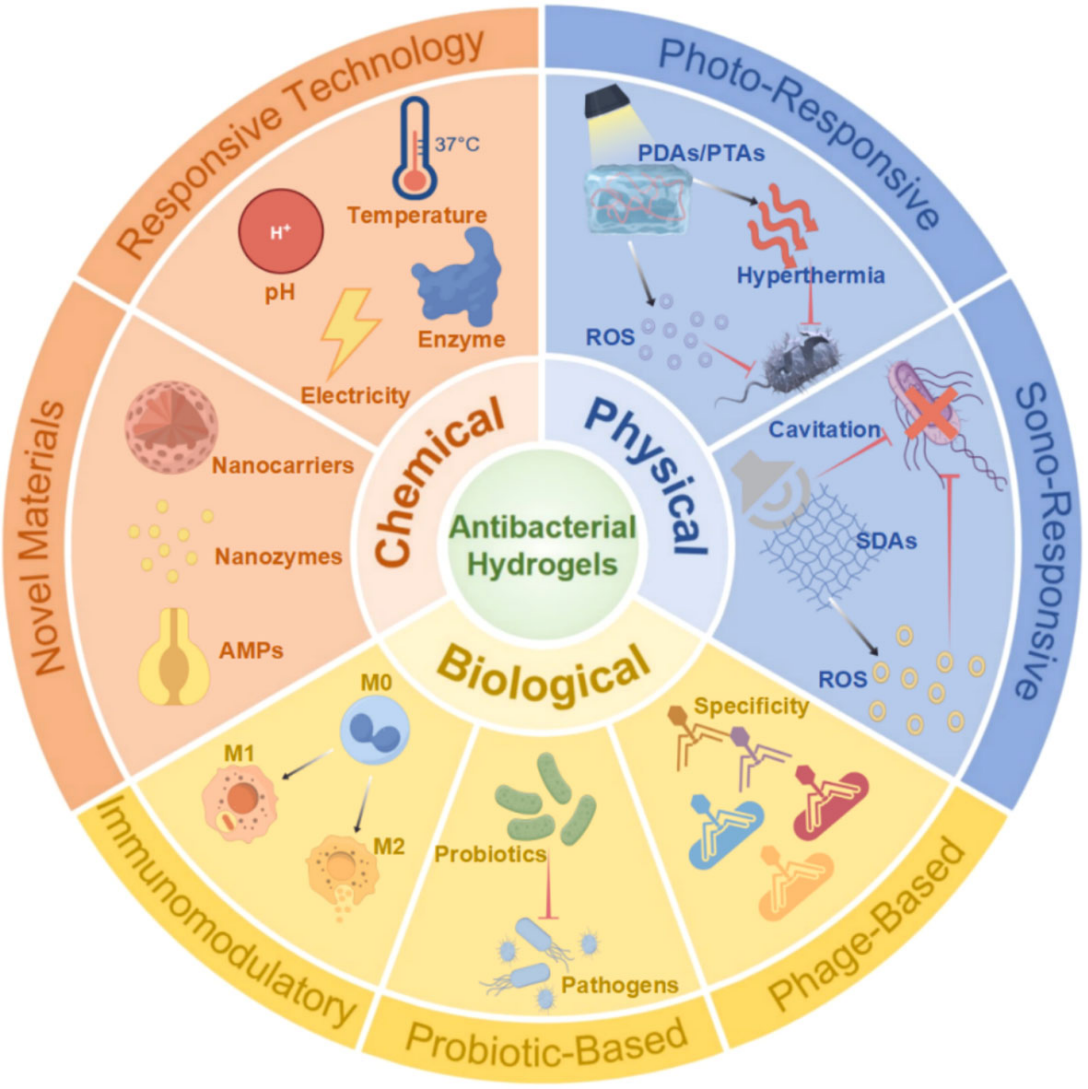
Schematic illustration of antibacterial hydrogels classified by chemical, physical, and biological mechanisms for infected wound management. Created with Figdraw (by figdraw.com). Used with permission for journal publication only.

Antibacterial hydrogels are a class of hydrophilic polymeric materials featuring three-dimensional crosslinked networks that, beyond their exceptional water retention, biodegradability, and superior tissue compatibility, function as versatile drug carrier platforms enabling controlled release and they have gained widespread clinical application in the treatment and management of infected wounds. (Li et al., 2018; Su et al., 2024) In contrast to systemic antibiotic administration, antibacterial hydrogels enable localized, sustained, and effective antimicrobial therapy while simultaneously promoting tissue regeneration. (Zhang et al., 2024c) From a mechanistic perspective, existing antibacterial hydrogels are principally categorized into three strategies: chemical, physical, and biological antibacterial approaches (**Figure 1**). These complementary therapeutic modalities synergistically establish a novel paradigm for the comprehensive management of infected wounds.

This review comprehensively consolidates the latest advances in antibacterial hydrogels for treating skin-infected wounds, highlighting the fundamental principles, material design, and overall efficacy of the three antimicrobial mechanisms, while further critically discussing the pivotal breakthroughs and current limitations. Through the evaluation of the advantages and challenges associated with various therapies, this work seeks to offer theoretical foundations and design concepts for the development of next-generation antibacterial hydrogels, thereby facilitating their translation from fundamental research to clinical applications. To facilitate quick cross-comparison, we summarized the core antibacterial mechanisms, key advantages, and primary translational challenges across these hydrogel strategies in **Table 1**.

**Table 1:** Summary of antibacterial hydrogel strategies for skin infected wounds regarding their core mechanisms, key advantages, and primary challenges.

Strategies		Core antibacterial mechanisms	Key advantages	Primary challenges
Chemical Antibacterial Hydrogels	Natural antibacterial materials	Electrostatic membrane disruption; quorum-sensing/biofilm inhibition; membrane lysis antimicrobial peptides (AMPs); sometimes immunomodulation	Strong biocompatibility; environmentally friendly; lower propensity to induce resistance (esp. AMPs)	Insufficient bactericidal potency may require higher doses; complex extraction/purification with high cost/low yield; stability issues from enzymatic degradation
	Synthetic antibacterial materials	ROS-mediated oxidative stress; metal-ion associated damage; multi-target killing (often tunable)	High antibacterial potency; improved durability; tunable structures and multifunctionality	Biodegradation pathways and long-term tissue retention/toxicity may be unclear; limited clinical validation; regulatory and safety evaluation requirements
	Stimulus-responsive hydrogels	Microenvironment-triggered activation/release for on-demand antibacterial action	Better spatiotemporal control; potential to reduce off-target exposure; supports precision therapy	Wound stimuli vary spatially/temporally, weakening specificity; limited sensitivity to weak stimuli; structural fatigue/performance degradation after repeated cycles
Physical Antibacterial Hydrogels	Photo-responsive hydrogels	Light-triggered hyperthermia photothermal therapy (PTT) and/or ROS generation photodynamic therapy (PDT) to kill bacteria/biofilms	Broad-spectrum action including drug-resistant pathogens; strong controllability (wavelength/intensity)	Limited light penetration and coverage area; equipment needs/cost; thermal discomfort/tissue damage risk; may require repeated sessions
	Sono-responsive hydrogels	Ultrasound-induced cavitation/mechanical disruption; synergistic ROS generation sonodynamic therapy (SDT); ultrasound-triggered drug release	Non-invasive; deeper tissue penetration than light; potentially cost-effective	Parameter optimization needed to balance efficacy/safety; dependence on ultrasound equipment; reactive oxygen species (ROS) overproduction must be controlled to avoid host damage
Biological Antibacterial Hydrogels	Immunomodulatory hydrogels	Modulates host immune response (e.g., orchestrating inflammatory-to-pro-repair transitions)	Addresses infection + inflammation + healing together; antibiotic-sparing potential	Macrophage states are dynamic and incompletely defined; lack of standardized classification; precise phenotype control remains difficult
	Probiotic-based hydrogels	Competitive exclusion; metabolite/bacteriocin production (e.g., lactic acid, reuterin); supports microbiome balance	Antibiotic-free; preserves/repairs microbial homeostasis; may reduce dysbiosis	Safety/controllability of live bacteria (secondary infection risk); strong regulatory/ethical oversight; maintaining viability during storage/transport is challenging
	Bacteriophage-based hydrogels	Host-specific phage lysis; localized sustained delivery; enhanced biofilm penetration in engineered systems	High specificity; effective against multidrug resistance (MDR) strains; self-amplifying at the infection site	Instability/immune clearance; biofilms can limit efficacy; narrow host range; bacterial anti-phage resistance; complex pharmacokinetics/pharmacodynamics (PK/PD) for translation

AMPs, antimicrobial peptides; ROS, reactive oxygen species; PTT, photothermal therapy; PDT, photodynamic therapy; SDT, sonodynamic therapy; MDR, multidrug resistance; PK/PD, pharmacokinetics/pharmacodynamics.

## 2 Chemical antibacterial hydrogels

Chemical antibacterial hydrogels exert their effects by incorporating diverse natural or synthetic antimicrobial agents within the hydrogel matrix to achieve chemical eradication of bacteria. Furthermore, through intelligent responsive designs, these hydrogel systems can modulate the release of incorporated compounds, exhibiting sustained, localized antibacterial activity. This approach minimizes systemic side effects, enhances therapeutic precision, and helps mitigate the growing problem of bacterial resistance (Liu and Shen, 2025; Wang et al., 2022).

### 2.1 Natural antibacterial materials

Numerous naturally occurring substances with intrinsic antimicrobial properties, derived from plants or animals, have attracted extensive attention in the field of antibacterial materials due to their favorable biocompatibility and relatively low cytotoxicity. (Zhou et al., 2024) Chitosan, an abundantly available and high-performance natural linear cationic polysaccharide, is primarily composed of D-glucosamine (GlcN) and N-acetyl-D-glucosamine (GlcNAc) residues, typically obtained by the deacetylation of chitin extracted from crustacean exoskeletons. (Tan et al., 2022) The positively charged amino groups (-NH_2_) along its molecular backbone interact electrostatically with negatively charged bacterial cell membranes, compromising membrane integrity and inducing bacterial death through cytoplasmic leakage. (Chang et al., 2020) Natural polyphenols represent another essential category of plant-derived antibacterial constituents, widely distributed in fruits, vegetables, tea leaves, and traditional medicinal herbs, with notable representatives encompassing tannic acid, curcumin, quercetin, and resveratrol. (Song et al., 2021; Zhang et al., 2023a; Tang et al., 2025). These compounds efficiently inhibit bacterial adhesion and motility while disrupting quorum sensing among bacterial communities to suppress biofilm formation. (Gonçalves et al., 2023) Yet these natural antibacterial substances still encounter limitations in practical applications owing to insufficient bactericidal potency, typically requiring elevated concentrations to accomplish meaningful anti-pathogenic outcomes, which in turn constrains their clinical translation prospects.

Recent years have witnessed remarkable breakthroughs in antimicrobial peptide research that have infused novel vigor into the development of antibacterial hydrogels. Antimicrobial peptides (AMPs), a heterogeneous group of small-molecule peptides with broad origins and varied structural configurations, typically comprise 10 to 50 amino acid residues and are ubiquitously present in plants, animals, and microorganisms as integral components of the innate immune defense system. (Ciobanasu et al., 2024) Endowed with pronounced cationic characteristics attributed to their high content of basic amino acids (such as lysine and arginine), AMPs can actively and firmly bind to the negatively charged bacterial cell membranes, then insert into the phospholipid bilayer via their hydrophobic regions, creating nanopores that ultimately lead to bacterial lysis due to loss of membrane integrity and efflux of intracellular contents. (Li et al., 2019; Salama et al., 2021; Sun et al., 2019; Oliveira Júnior et al., 2025) It is this direct physical disruption mechanism targeting cellular membranes that renders bacteria less susceptible to developing resistance mutations (Xiao et al., 2025; Zhao et al., 2025a). Zhou et al. (2023) developed an injectable self-assembling hydrogel with natural antimicrobial peptide *Jelleine-1* that achieved >98.5% killing efficacy against *methicillin-resistant Staphylococcus aureus* (MRSA), *E. coli*, and *C. albicans in vitro* without the need for chemical crosslinkers. Notably, the system circumvents bacterial resistance through membrane-lytic mechanisms while maintaining excellent biocompatibility. Additionally, AMPs are capable of enhancing host resistance against pathogens by modulating immune responses and activating macrophages (Xiang et al., 2021; Zhen et al., 2022). Liu et al. (2023) created a supramolecular antimicrobial peptide hydrogel (C12G2) using alkylated α-helical peptide *C12-G(IIKK)2I-NH2*, which combines membrane disruption with immunomodulation for enhanced bactericidal activity. AMPs are recognized as one of the most promising antibiotic alternatives for future anti-infective therapy because of their broad-spectrum activity, high efficacy, low toxicity, and low propensity to induce bacterial resistance, particularly valuable against multidrug-resistant strains (Oliveira Júnior et al., 2025).

Despite their exceptional biocompatibility and environmental sustainability, natural antibacterial materials face considerable challenges in real-world clinical applications. Their complex extraction and purification requirements, combined with limited raw material sources, result in low yields and high costs that hinder large-scale commercial manufacturing. Moreover, these materials suffer from poor stability due to their susceptibility to endogenous enzymatic degradation, undermining their long-term antimicrobial effectiveness. Consequently, strategies such as molecular structural modification or nanocarrier encapsulation are critical approaches to address these fundamental problems.

### 2.2 Synthetic antibacterial materials

To address the constraints of natural antibacterial materials, researchers have intensified the development of synthetic antibacterial materials that integrate potent antibacterial efficacy, outstanding biocompatibility, and enhanced durability. Unlike their natural counterparts, synthetic systems not only deliver excellent long-term stability and cost-effectiveness but, more importantly, are characterized by precisely tunable molecular structures that enable targeted performance optimization. Specifically: (1) the design of multi-target mechanisms can amplify antimicrobial activity, realizing broad-spectrum bacterial killing and preventing the emergence of resistance; (2) biomimetic modifications bestow superior tissue compatibility to fulfill extended clinical requirements for cutaneous wound healing without eliciting detrimental immune reactions.

#### 2.2.1 Metal and metal oxide nanozymes

The swift progress in nanomaterials has positioned metal and metal oxide nanozyme particles as key research targets in antibacterial materials research. These nanostructures, distinguished by dimensions below 100 nm and extraordinary specific surface areas, demonstrate significantly enhanced surface reactivity and biological activity relative to traditional inorganic metallic ones. (Krebsz et al., 2021; He et al., 2016) Their size and structural benefits dramatically improve interaction efficiency with bacterial cells, allowing effortless membrane penetration and cytoplasmic entry. (He et al., 2016) Most importantly, these nanoparticles display enzyme-mimetic catalytic capabilities, replicating natural enzyme functions (including peroxidase and oxidase) under appropriate substrate conditions to produce substantial reactive oxygen species (ROS) with high oxidative potential, such as superoxide anions (O_2_^-^) and hydroxyl radicals (·OH). (Wang et al., 2025a, 2021) These ROS molecules rapidly initiate cascades involving lipid peroxidation, protein denaturation, and DNA fragmentation, bringing about bacterial elimination via oxidative stress (Tikhomirova et al., 2024).

**Figure 2:**
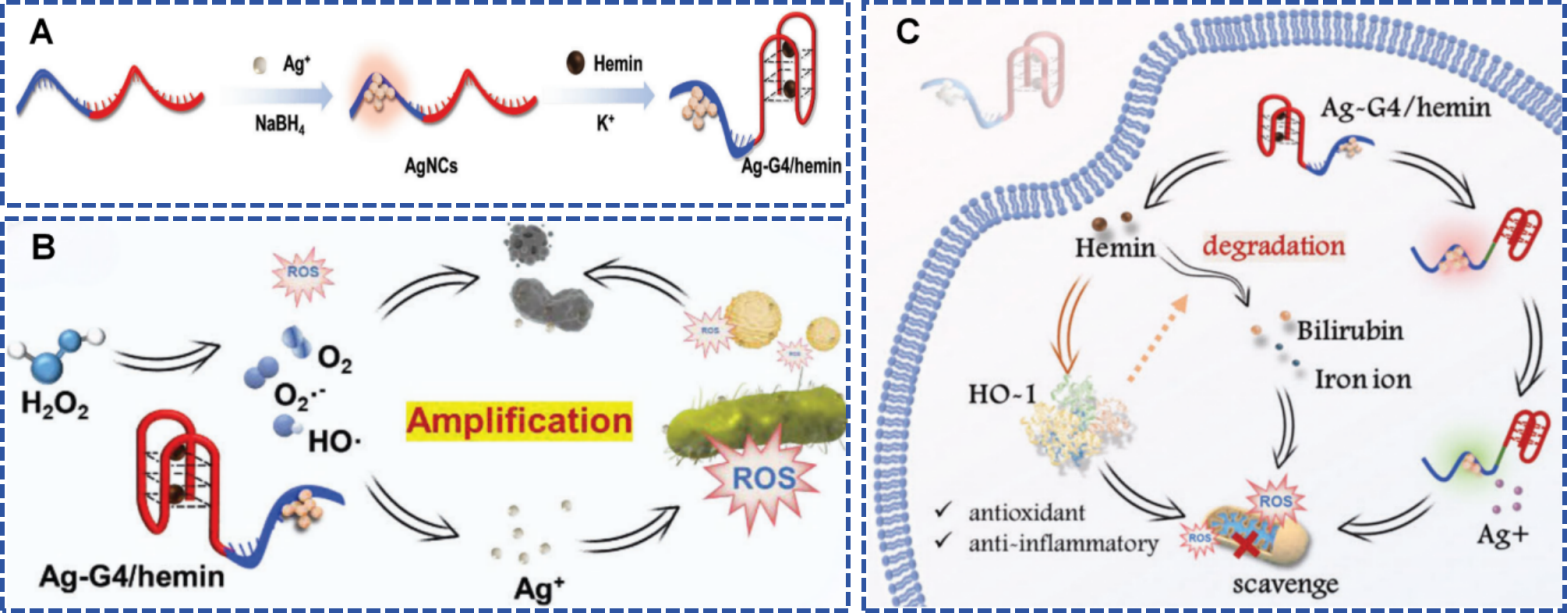
Diagram depicting **(A)** the construction of Ag-G4/hemin nanoclusters and **(B, C)** their synergistic antibacterial and cellular protective mechanisms. Reproduced with permission (Zhu et al., 2023). ^©^ 2023 Wiley-VCH GmbH. All rights reserved.

Silver stands as the quintessential antibacterial metal, having undergone thorough investigation and widespread clinical application in infection control. Although evidence suggests minimal direct cytotoxic effects on mammalian cells at therapeutic doses, (Jia et al., 2023) its poor biodegradability raises major concerns regarding cumulative toxicity following prolonged exposure. (Singer and Bourauel, 2023) Zhu et al. (2023) engineered a sophisticated hydrogel wound dressing featuring G-quadruplex/hemin (G4/hemin) DNAzyme-modified silver nanoclusters (Ag-G4/hemin). This system utilizes bifunctional DNA scaffolds where cytosine-dense sequences template silver nanocluster formation, while guanine-rich domains coordinate K^+^ to assemble G4/hemin complexes, conferring concurrent peroxidase (POD) and catalase (CAT) activities (**Figure 2A**). In infected wound microenvironments, G4/hemin harnesses its POD capability to transform hydrogen peroxide (H_2_O_2_) into potent hydroxyl radicals while exploiting its CAT function to convert surplus H_2_O_2_ to O_2_, thus promoting nanocluster oxidation and Ag^+^ release (**Figure 2B**). This dual-action mechanism eliminated 99.9% of MRSA, *S. aureus*, and *E. coli*, and animal studies revealed a five-log bacterial reduction within 24 hours in MRSA-infected wounds. Crucially, the physiological H_2_O_2_ levels in normal tissues resulted in Ag^+^ release rates that were one-eighth of those at infection foci, while G4/hemin substantially mitigated Ag^+^-mediated oxidative toxicity via ROS neutralization and heme oxygenase-1 activation (**Figure 2C**). This work seamlessly merges silver’s antimicrobial potency with DNAzyme catalytic precision, overcoming traditional silver dressing drawbacks of cytotoxicity and limited functionality, thus delivering a smart therapeutic platform that balances robust pathogen elimination with superior biocompatibility for multidrug-resistant infections.

**Figure 3:**
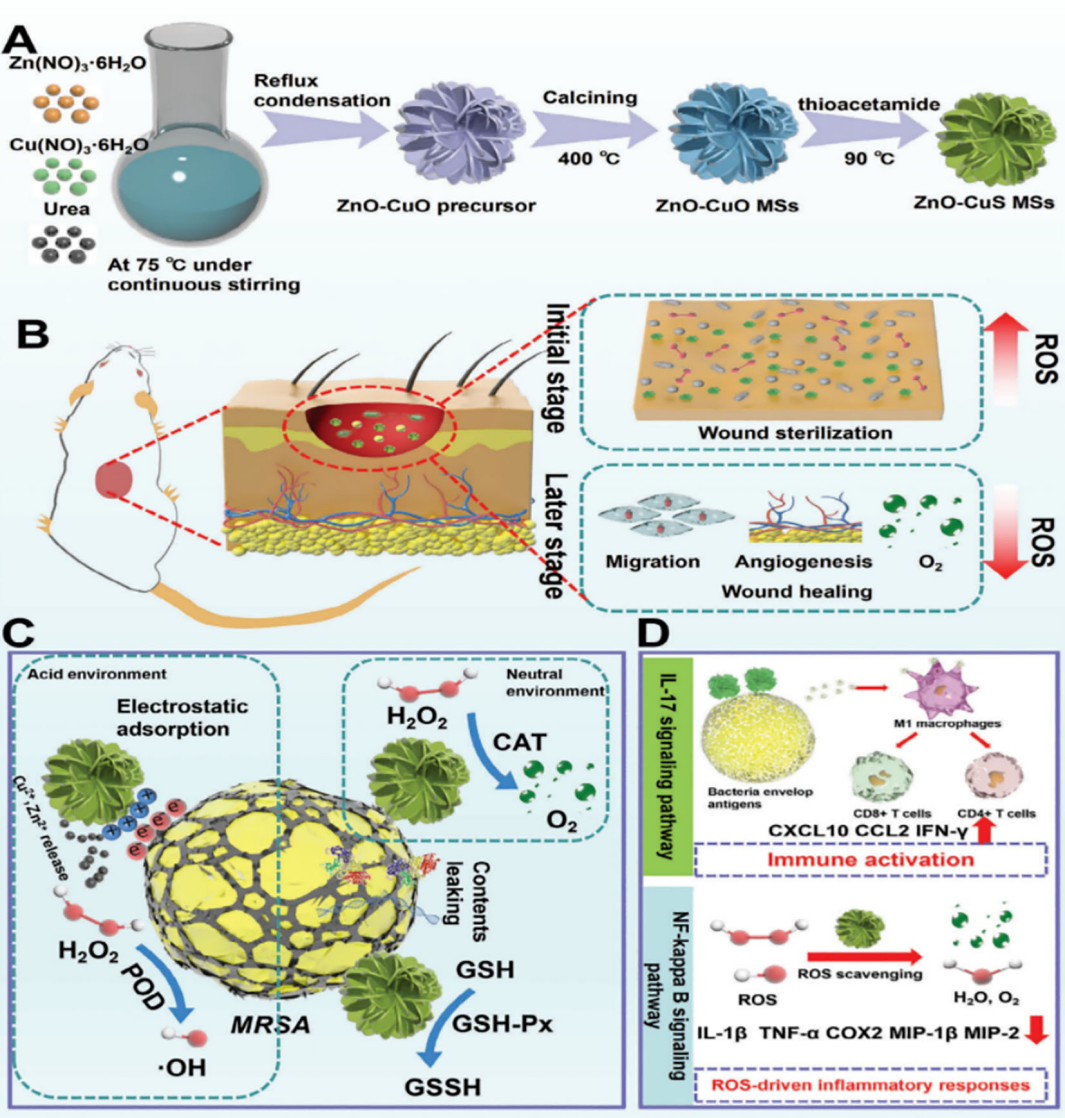
**(A)** The synthesis route and **(B–D)** multifunctional mechanisms of ZnO–CuS nanozymes in promoting the healing of infected wounds. These nanozymes integrate antibacterial activity, oxidative modulation, and inflammatory regulation to accelerate tissue repair. Reproduced with permission (Zhang et al., 2024a). ^©^ 2024 Wiley-VCH GmbH. All rights reserved.

In contrast, as a vital trace element essential for biological functions and a cofactor in numerous transcriptional and enzymatic processes, zinc has demonstrated exceptional biocompatibility and tissue-repair-promoting properties, making it a preferred material in tissue engineering (Jia et al., 2023; Hoang et al., 2020; Zhao et al., 2024). Zhang et al. (2024a) developed zinc oxide-reinforced copper sulfide nanozyme microspheres (ZnO-CuS MSs) that deliver dual antibacterial and anti-inflammatory therapy for infected wounds via pH-triggered transitions in enzymatic activity (**Figure 3A**). The composite framework harnesses ZnO and CuS properties, embodying peroxidase-mimetic function under acidic infection conditions to eliminate pathogens, then converting to catalase-mimetic behavior as the wound pH normalizes to neutralize excess ROS while concurrently dampening inflammatory mediator production (**Figures 3B–D**). Unlike traditional nanozymes, this platform uniquely combines metal oxide and sulfide to achieve synergistic effects, facilitating temporal control over the “sterilization-healing” process through pH-mediated enzymatic modulation.

#### 2.2.2 Peroxide-based nanocarriers

Established as a conventional disinfectant, H_2_O_2_ has garnered increasing scientific interest for its therapeutic promise in accelerating tissue repair beyond its antibacterial capacity. Studies indicate that H_2_O_2_ at appropriate concentrations serves as a crucial mediator in wound repair mechanisms, acting as a bioactive signaling molecule that orchestrates leukocyte migration and cytokine secretion, subsequently stimulating epithelial cell proliferation, angiogenesis, and tissue regeneration. (Bilgen et al., 2019; Dunnill et al., 2017) Laboratory findings show that 10 μM H_2_O_2_ acts as a chemoattractant to direct T-cell accumulation toward injury sites via pathways independent of circulating signals; 100 μM H_2_O_2_ promotes capillary formation by triggering signaling pathways associated with vascular endothelial growth factor (VEGF), thereby improving blood perfusion in wound regions. (Dunnill et al., 2017) However, maintaining precise H_2_O_2_ dosing protocols is essential, since excessive levels can intensify the ROS burden and counterproductively impair recovery in infected skin lesions (Huang et al., 2022).

**Figure 4:**
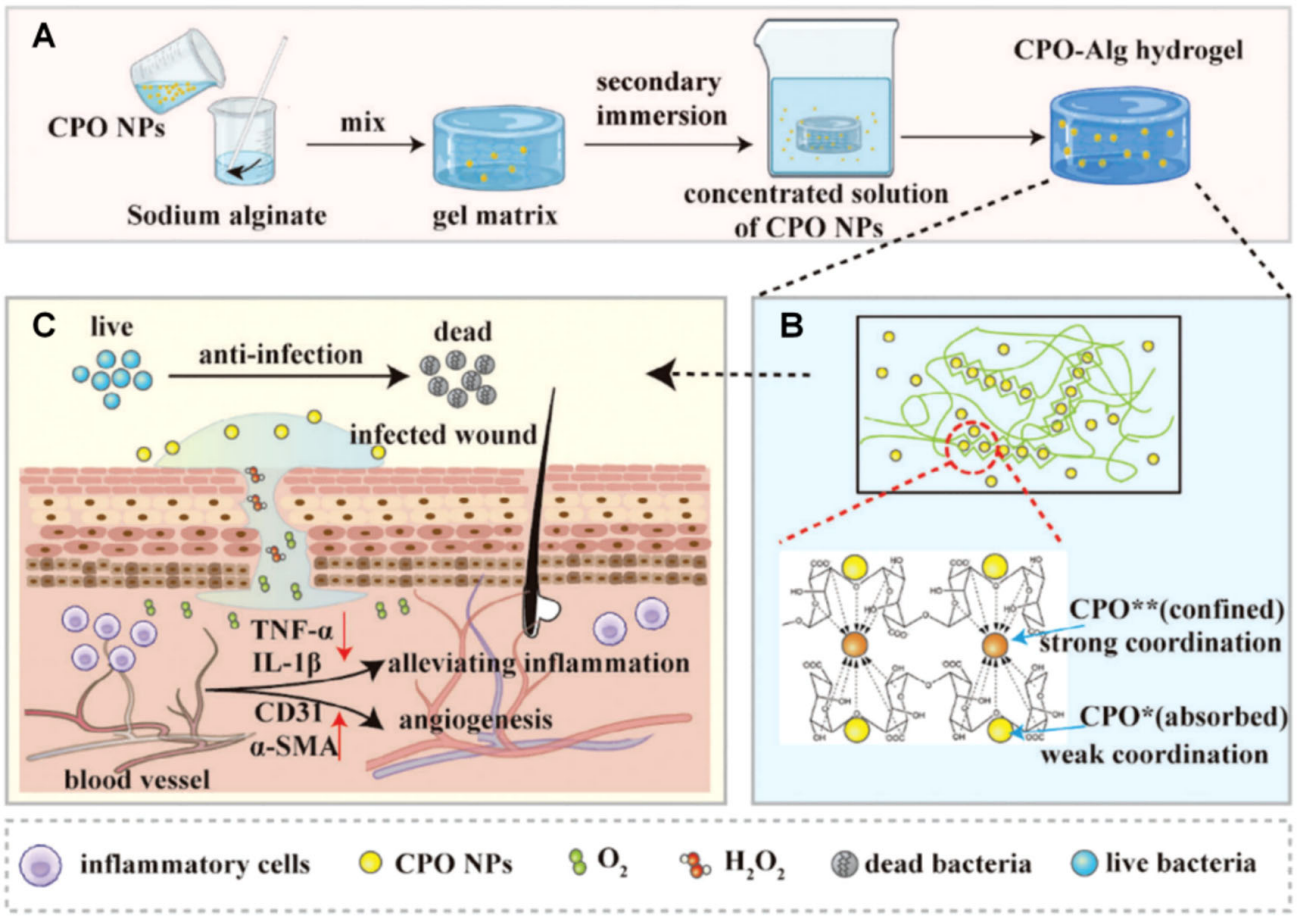
Schematic illustration of **(A)** the preparation process and **(B, C)** therapeutic mechanism of CPO-Alg hydrogels for bacteria-infected wound healing. The CPO-Alg hydrogels are fabricated through a two-step coordination strategy that enables the coexistence of calcium peroxide nanoparticles in distinct binding states within the gel network. Reproduced with permission (Huang et al., 2024b). ^©^ 2024 The Authors. All rights reserved. Published by Wiley-VCH GmbH under the Creative Commons CC BY license.

Building on these findings, Huang and colleagues (Huang et al., 2024b) developed an intelligent antibacterial hydrogel system (CPO-Alg) based on calcium peroxide nanoparticles (CPO NPs) and alginate (Alg), fabricated through a two-step synthesis method to construct three-dimensional network structures via electrostatic interactions and ionic crosslinking (**Figure 4A**). Within this system, loosely-bound CPO NPs distributed throughout the hydrogel pores provide rapid and high-concentration H_2_O_2_ release for early-stage infection control, while structurally-integrated CPO NPs embedded in the network framework as crosslinking nodes allow sustained and low-concentration H_2_O_2_ release for tissue repair (**Figure 4B**). This distinctive architecture confers precise control over the release kinetics of CPO NPs to attain on-demand H_2_O_2_ liberation. *In vitro* studies showed >99.99% bactericidal efficacy against *E. coli* and *S. aureus* during early infection, followed by enhanced fibroblast migration and neovascularization amid later healing phases (**Figure 4C**). Animal studies revealed significant bacterial reduction in infected wounds within 24 hours, 100% wound closure within 14 days, and a 66.7% increase in collagen deposition versus controls.

#### 2.2.3 Novel co-assembly superstructures

**Figure 5:**
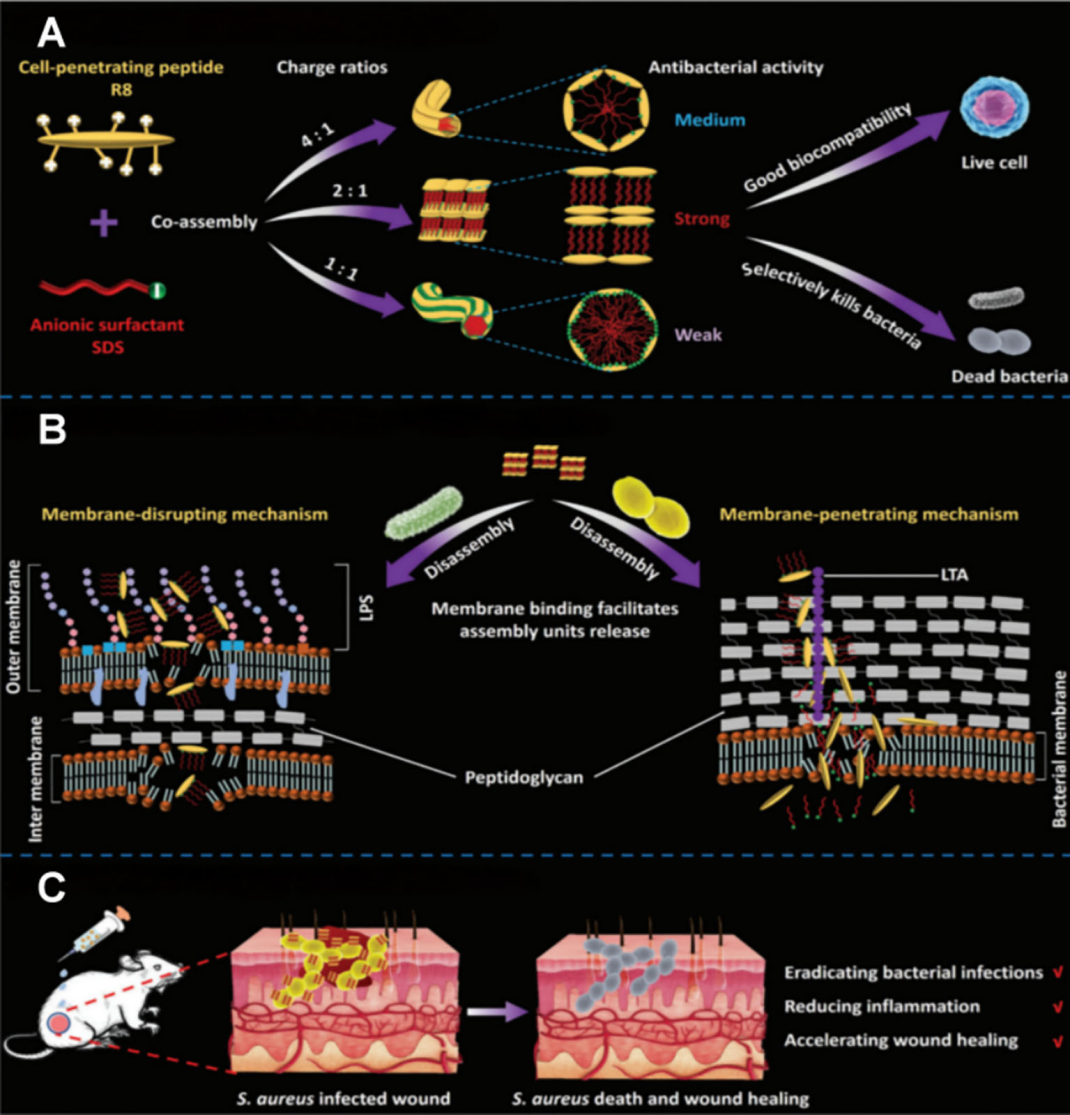
**(A)** Co-assembly and bioactivity of R8/SDS. **(B)** Antibacterial mechanism of R8/SDS lamellar structure. These superstructures eradicate *E*. *coli* and *S. aureus* via membrane disruption and membrane penetration, respectively. **(C)** *In vivo* application and performance of R8/SDS. Reproduced with permission. (Gong et al., 2024) ^©^ 2024 Wiley-VCH GmbH. All rights reserved.

Recently, Gong et al. (2024) discovered that two molecules lacking intrinsic antibacterial activity could present remarkable bactericidal properties when combined synergistically. Octa-arginine (R8), a cell-penetrating peptide composed of eight consecutive arginine residues, shows negligible bacterial inhibition alone. Sodium dodecyl sulfate (SDS), an anionic surfactant, exhibits only limited antimicrobial efficacy at conventional concentrations. The research team designed a supramolecular co-assembly system with adjustable charge ratios by combining R8 and SDS through electrostatic and hydrophobic interactions. At a 2:1 charge ratio, strong complementarity between arginine’s guanidinium groups and sulfate moieties forms lamellar structures that manifest maximal antibacterial activity, improving antibacterial efficiency by nearly 100-fold compared with the individual components (**Figure 5A**). This system employs distinct mechanisms for different bacterial types: against Gram-negative bacteria (such as *E. coli*), insertion of hydrophobic alkyl chains located at lamellar structure edges into the lipopolysaccharide membrane results in the structural disruption and destruction; for Gram-positive bacteria (such as *S. aureus*), R8’s penetrating properties enable electrostatic interactions with negatively charged ladder-like lipoteichoic acids that longitudinally traverse the peptidoglycan layer, forming transmembrane channels that allow SDS to enter the cytoplasm and denature intracellular proteins (**Figure 5B**). Animal studies confirmed that the system creates targeted antimicrobial barriers at wound sites while releasing arginine from its degradation products to promote ordered collagen arrangement and markedly accelerate tissue repair (**Figure 5C**). This work pioneers an understanding of how non-antibacterial components achieve synergistic bactericidal effects through supramolecular engineering, thereby establishing design frameworks for next-generation anti-infective materials.

Despite the significant advantages that synthetic antibacterial materials offer in anti-infective therapy, their clinical translation faces substantial obstacles. Currently, the biodegradation pathways of most synthetic compounds remain incompletely characterized, with certain materials and their metabolic derivatives potentially undergoing only partial degradation, creating risks of prolonged tissue retention and consequent toxicity. Moreover, most current research remains at the preclinical stage and lacks rigorous validation through extensive clinical studies, necessitating thorough assessment of long-term efficacy and safety profiles. Therefore, future investigations must comprehensively examine biodegradation kinetics, biocompatibility parameters, and clinical viability for extended applications while preserving antimicrobial potency. Meanwhile, developing holistic assessment protocols and establishing transparent regulatory frameworks for clinical authorization will be crucial for advancing their successful implementation in healthcare settings.

### 2.3 Stimulus-responsive hydrogels

As novel antibacterial materials continue to emerge, the escalation of precision medicine and growing demand for personalized therapy have underscored the clinical importance of innovative drug delivery systems. Especially in the context of increasingly severe antimicrobial resistance, achieving intelligent control and precise drug delivery has become a key research focus. Stimulus-responsive hydrogels, also called smart or adaptive hydrogels, are three-dimensional polymer networks that respond reversibly to external stimuli such as pH, temperature, enzymes, electric fields, light irradiation, and acoustic waves. (Liu and Shen, 2025; Phan et al., 2017; Zhao et al., 2023) These hydrogels are characterized by their environmental adaptability and dynamic regulation, they undergo volume changes or gel-solution transitions under specific stimuli, securing on-demand drug release and targeted delivery. (Zhao et al., 2023) Such systems improve treatment precision and timing while reducing drug waste and systemic side effects, thereby lowering the risk of the evolution of bacterial resistance (Hao et al., 2024).

#### 2.3.1 pH-responsive hydrogels

The pH-responsive mechanism represents one of the most prominent anti-infective strategies in stimulus-responsive hydrogel systems. Healthy skin maintains a mildly acidic microenvironment (pH 4.0-6.0) through sebaceous and sweat gland secretions, forming an essential component of the natural skin barrier (Tsegay et al., 2022) However, following the barrier compromise and subsequent pathogenic invasion, the local wound pH would undergo dynamic alterations. (Municoy et al., 2020; Youssef et al., 2023) During early acute infection, inflammatory cell infiltration coupled with active bacterial growth collectively generate many acidic metabolites, causing a transient decline in local environmental pH. (Municoy et al., 2020; Youssef et al., 2023; Barnum et al., 2020) If infection persists, disease progression and wound chronicity would give rise to the accumulation of alkaline bacterial byproducts, combined with chronic inflammatory exudation, ultimately elevating wound pH to 7.2-8.9 or even higher. (Youssef et al., 2023; Morán et al., 2024) Such characteristic pH variation during wound infection underlies the rationale for developing pH-responsive hydrogels.

Many pH-responsive hydrogels incorporate ionizable groups into polymer chains to realize pH sensitivity via reversible protonation and deprotonation reactions under varying pH conditions, such as acidic groups (-SO_3_H, -COOH) or basic groups (-NH_2_). (Rando et al., 2024; Dsouza et al., 2022) For instance, Xiong et al. (2024) invented a multifunctional pH-responsive hydrogel (CPT) using carboxymethyl chitosan (CMCS), polyvinylpyrrolidone (PVP), and tannic acid (TA) as crosslinkers for treating drug-resistant bacterial infections. The carboxyl groups in CMCS deliver exceptional pH sensitivity: at pH 5.5, the protonated carboxyl groups (-COOH) result in hydrogel contraction; at pH 7.4-8.5, the deprotonated carboxylate ions (-COO^-^) would generate electrostatic repulsion to trigger network expansion. This structural transformation directly influences the hydrogel’s porous architecture and crosslinking density, leading to a 3.2-fold increase in swelling ratio and establishing pathways for drug release. The smart swelling-shrinking behavior enables rapid TA release in the alkaline microenvironment, manifesting antioxidant activity and broad-spectrum antibacterial efficacy against MRSA and *meropenem-resistant E. coli*.

Beyond designs based on physical responsiveness via ionization reactions, another prevalent strategy involves constructing pH-sensitive reversible chemical bonds, such as imine bonds (also known as Schiff base bonds) or acylhydrazone bonds. (Wu et al., 2024; Brito et al., 2021) Imine bonds are typically formed through dehydration reactions between primary amines and aldehydes or ketones, (Araujo de Oliveira et al., 2022; Lee, 2018) while acylhydrazone bonds result from reactions between carbonyls and hydrazine derivatives (Mahmoudi et al., 2024). Hydrogel degradation and drug release occur when these chemical bonds are hydrolyzed under acidic conditions (Serpico et al., 2023). Jin et al. (2024) have innovated a nanofiber-reinforced self-healing polysaccharide hydrogel dressing (T/B@PLA/OSA/CMCS) that integrates pH visualization and dynamic controlled drug release. The hydrogel creates a three-dimensional porous structure through crosslinking oxidized sodium alginate (OSA) and carboxymethyl chitosan (CMCS) by dynamic Schiff base bonds and hydrogen bonding, with polylactic acid (PLA) nanofibers for mechanical reinforcement. Bromothymol blue (BTB) indicator changes from blue to yellow in infected environments (pH 4.5-6.0) for visual monitoring, while Schiff base bond hydrolysis would accelerate tetracycline hydrochloride (TH) release to kill pathogens. This system presents an intelligent management solution that combines both diagnostic and therapeutic capabilities for chronic infected wounds, with synergistic responsive monitoring, on-demand drug delivery, and mechanical improvement.

#### 2.3.2 Temperature-responsive hydrogels

Temperature-responsive hydrogels are intelligent wound dressings capable of reversible phase transitions in response to temperature variations. (Peng et al., 2022) Poly(N-isopropylacrylamide) (PNIPAM) is one of the most extensively investigated thermosensitive materials used to fabricate such hydrogels, which display a characteristic lower critical solution temperature (LCST) behavior. (Tan et al., 2021) The LCST is defined as the critical temperature below which a polymer and solvent form a homogeneous single-phase solution; above this temperature, intensified hydrophobic interactions between polymer chains cause phase separation. (Theodoropoulou et al., 2024) PNIPAM’s LCST of approximately 32 °C closely matches human body temperature, making it ideal for biomedical applications. (Narayana et al., 2025; Deng et al., 2023; Li et al., 2023) Below the LCST, the hydrophilic amide groups within PNIPAM molecules form extensive hydrogen bonding with water molecules, inducing swelling of the entire polymer network and maintaining the hydrogel in a highly hydrated and hydrophilic state (Deng et al., 2023; Li et al., 2023). Under these conditions, the loose network structure can facilitate gradual drug release, thus achieving sustained therapeutic efficacy. Above the LCST, hydrogen bonding is disrupted, exposing hydrophobic isopropyl groups and sharply intensifying hydrophobic interactions between polymer chains, leading to abrupt hydrogel contraction or gel-solution phase transition (Deng et al., 2023; Li et al., 2023). The resulting compression swiftly expels encapsulated drugs from the gel matrix, making this system advantageous for applications requiring immediate increases in drug concentration. In essence, the thermoresponsive behavior stems from the dynamic equilibrium between hydrophilic and hydrophobic group interactions within the polymer structure, where temperature fluctuations alter this equilibrium, driving reversible hydrophilic-hydrophobic transitions that enable controlled drug release.

Recently, Bai et al. (2024) built on traditional thermosensitive hydrogels to address their inherent limitation of a sluggish response to infection dynamics, engineering an innovative anti-infective microneedle patch that integrates temperature sensing, wireless monitoring, and intelligent drug release. The patch employs a porous chitosan matrix loaded with PNIPAM and embedded sensor chips equipped with Bluetooth modules to create a closed-loop temperature-drug release system. When wound temperature exceeds 36.5 °C for 6 hours, the microcontroller triggers heating elements that contract PNIPAM, precisely releasing encapsulated rifampicin. In animal models, the patch attained real-time wound temperature monitoring with high precision (± 0.5 °C) and enabled multiple subsequent drug-release episodes, reaching 91.6% wound healing by day 9, which significantly outperformed conventional passive-release dressings.

#### 2.3.3 Enzyme-responsive hydrogels

Enzymes constitute indispensable functional biomolecules that participate in virtually all biological processes within living organisms. Enzyme-responsive hydrogels leverage pathogen-secreted enzymes as triggers, capitalizing on the inherent efficiency and selectivity of enzymatic reactions to achieve enhanced responsiveness and sensitivity (Mu et al., 2018).

**Figure 6:**
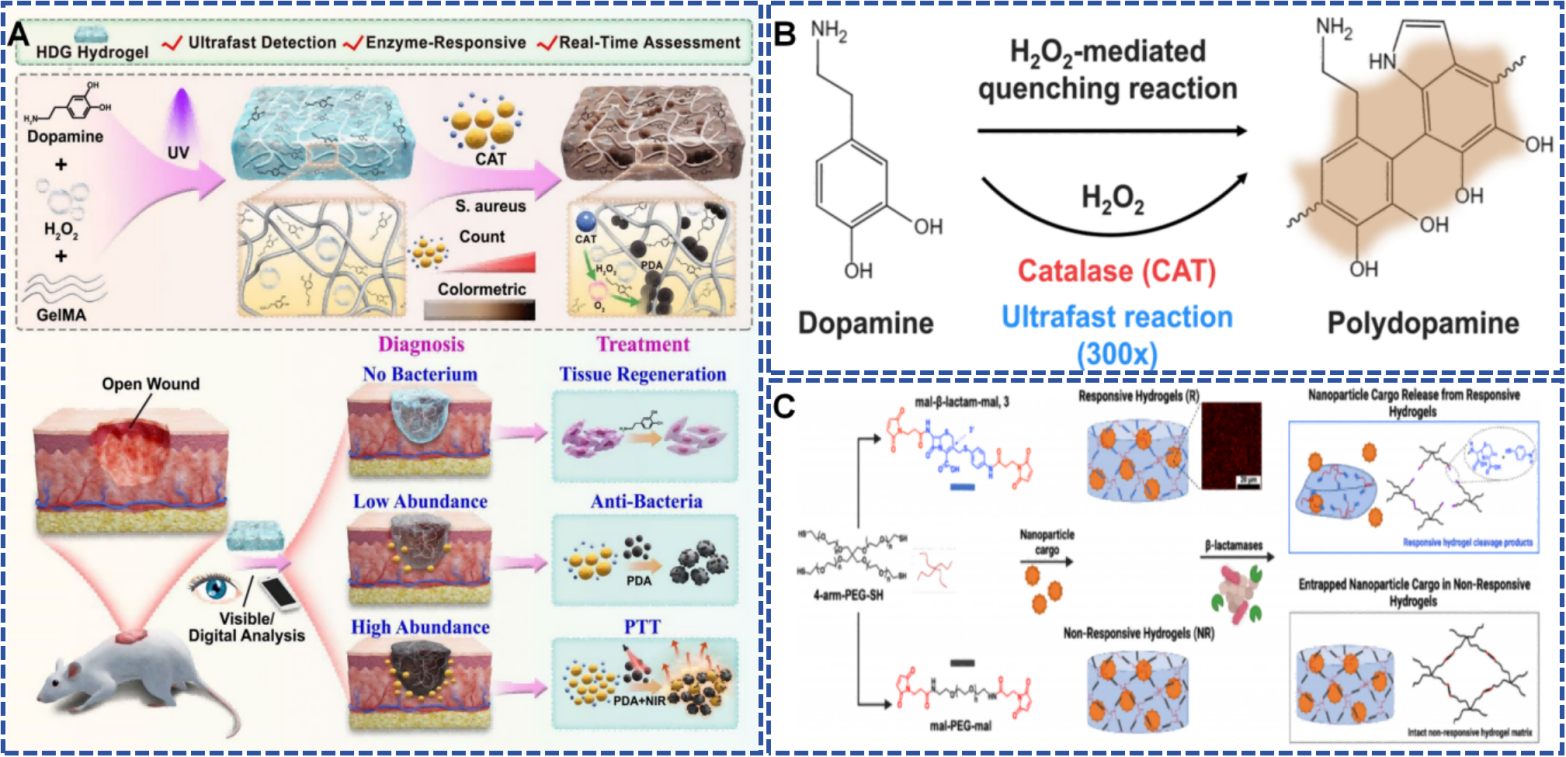
**(A, B)** Schematic diagrams showing the preparation and working mechanism of the HDG hydrogel, which integrates ultrafast enzyme-triggered polymerization and real-time infection monitoring for optimized wound treatment. At low bacterial loads, a strong antibacterial effect can be achieved solely through membrane disruption by polydopamine (PDA). At high bacterial loads, the photothermal therapy (PTT) becomes the preferred option as PDA generates photothermal effects under near-infrared (NIR) light. Reproduced with permission. (Zhao et al., 2025b) ^©^ 2025 The Authors. All rights reserved. Published by Springer Nature under the Creative Commons CC BY-NC-ND 4.0 license. **(C)** Illustration of a β-lactamase-responsive hydrogel platform fabricated via thiol-ene Michael-type addition, where enzymatic hydrolysis of the β-lactam bond triggers degradation of the hydrogel matrix and controlled release of nanoparticle cargo. Reproduced with permission. (Alkekhia et al., 2022) ^©^ 2022 American Chemical Society. All rights reserved.

As one of the central mechanisms by which bacteria respond to oxidative stress, CAT is a key enzyme that rapidly catalyzes the decomposition of H_2_O_2_ into water and oxygen. (Rasheed, 2024) Most common pathogenic bacteria are strongly CAT-positive, including *S. aureus*, *Pseudomonas*, *Burkholderia*, and *Serratia* species (Jesenak et al., 2014; Zhang et al., 2024b; Zhao et al., 2022). Hence the incorporation of H_2_O_2_ into hydrogel matrices to construct enzyme-driven, stimulus-responsive drug delivery systems holds considerable clinical potential. Zhao et al. (2025b) created an ultrafast enzyme-responsive hydrogel (HDG) containing H_2_O_2_, dopamine (DA), and gelatin methacrylate (GelMA), designed specifically for visual monitoring and targeted treatment of *S. aureus* infections (**Figure 6A**). When CAT from *S. aureus* catalyzes the breakdown of H_2_O_2_, the generated oxygen triggers DA polymerization within 10 minutes to form dark brown polydopamine and thereby produces an “enzyme-responsive” chromogenic reaction (**Figure 6B**). This process demonstrates high sensitivity, producing distinct color changes even at low bacterial loads to enable early infection detection. Furthermore, the extent of polydopamine formation correlates positively with bacterial concentration, providing intuitive visual assessment of infection severity. Therapeutically, the resulting polydopamine exhibits synergistic antibacterial effects by disrupting bacterial membranes and generating near-infrared photothermal effects, showing robust bactericidal activity against *S. aureus*.

In contrast to other bacterial secreted enzymes, β-lactamases represent a specific class of enzymes produced exclusively by bacteria that destroy the β-lactam ring structure prevalent among numerous commonly used antibiotics, constituting one of the primary mechanisms by which bacteria acquire drug resistance. (Tabcheh et al., 2023). Consequently, they are regarded as highly attractive bacteria-specific stimuli-responsive switches for drug delivery systems. Alkekhia et al. (2022) constructed a polyethylene glycol-based hydrogel platform that utilizes β-lactamase-responsive mechanisms for bacteria-triggered drug release. The system employs maleimide-functionalized cephalosporin derivatives as dynamic crosslinkers, forming three-dimensional networks through thiol-ene Michael-type addition with four-armed polyethylene glycol. When bacterial β-lactamases cleave amide bonds in the cephalosporin ring, the crosslinked structure dissociates, causing hydrogel disintegration and discharging encapsulated nanomedicine (**Figure 6C**). Notably, this drug delivery platform shows high selectivity for β-lactamase, with no response to other bacterial enzymes such as collagenase and lipase. *In vitro* experiments revealed that the hydrogel achieved complete degradation within 3.5 hours upon exposure to *P. aeruginosa*, with 93.1% drug release efficiency, and the release kinetics correlated linearly with hydrogel mass loss. Ex vivo porcine skin models confirmed the specificity of the response to multidrug-resistant bacteria, enabling wound monitoring and antibacterial delivery for 14 days. This approach cleverly exploits bacterial resistance mechanisms as triggers, offering innovative perspectives for intelligent drug delivery against drug-resistant infections.

#### 2.3.4 Electroresponsive hydrogels

Electroresponsive hydrogels capable of responding to external electric field stimuli consist of hydrogel matrices that incorporate conductive components. Commonly employed conductive materials include carbon nanotubes (CNTs), graphene oxide (GO), polypyrrole (PPy), polyaniline (PANI), and poly(3,4-ethylenedioxythiophene):polystyrene sulfonate (PEDOT: PSS). (Hao et al., 2024; Wang et al., 2024a; Li et al., 2024) However, these conductive components typically exhibit poor biocompatibility and therefore require combination with natural polyelectrolytes such as gelatin, chitosan, hyaluronic acid, and cellulose to improve biosafety and dispersion stability, thereby enhancing overall conductivity and gelation properties (Hao et al., 2024). Shi et al. (2025) created a multi-component noncovalently crosslinked conductive network by combining quaternized chitosan nanoemulsion microspheres (QCSNE) possessing electrostatic loading properties and PEDOT: PSS. Under low-voltage (1.5 V) electric field control, this hydrogel system induces migration and conformational rearrangement of charged groups in the matrix, triggering dynamic changes in physicomechanical properties. This process drives directional drug release opposite to the direction of charge migration, achieving rapid and controllable drug delivery. Experimental results showed broad compatibility with drugs of different molecular weights, including doxorubicin and rhodamine B, and demonstrated precise control over release timing and direction through adjustment of the electric field parameters.

Notably, electrical stimulation functions both as a controlled release trigger and as a therapeutic approach that reduces wound edema and accelerates tissue regeneration. (Raval et al., 2019; Zhang et al., 2023b) By activating specific ion channels and downstream signaling pathways, electrical currents modulate the expression of multiple genes critically involved in wound healing processes, promoting cell migration, proliferation, angiogenesis, and re-epithelialization (Raval et al., 2019; Zhang et al., 2023b; Nasra et al., 2024; Fu et al., 2023; Sun et al., 2024). Xiong et al. (2023) developed a polyvinyl alcohol-chitosan-graphene oxide (PCG) conductive hydrogel platform that enabled efficient transdermal delivery of sodium fluorescein (NaFL). Their *in vitro* studies showed that 2 V pulsed electrical stimulation significantly enhanced endothelial cell migration and increased VEGF secretion by 1.5-fold compared to controls, finally effectively facilitating vascular regeneration.

While stimulus-responsive hydrogels have displayed considerable promise for intelligent drug release and targeted antimicrobial therapy, several key barriers limit their clinical translation. The complex wound microenvironment presents multiple simultaneous stimuli, such as pH, temperature, and enzymes, that vary spatially and temporally, severely weakening the precision and specificity of hydrogel responses. Current systems also demonstrate insufficient sensitivity to weak stimuli, making precise control of drug release rates and dosages particularly challenging. In response to this issue, the potential strategies to enhance the specificity and sensitivity of stimuli-responsive hydrogels in complex wound environments involve multi-stimuli responsiveness, integrated sensing, and nanogel architectures. Multi-responsive hydrogels tuned to multiple triggers can dynamically adjust therapeutic release according to changing wound microenvironments, offering greater specificity and adaptive treatment than single-stimulus systems (Liu et al., 2025) Embedding biosensing components or integrating real-time monitoring systems further improves wound assessment and on-demand sense-and-treat responses, (Wang et al., 2023) while scale reduction to nanogel formats improves spatial resolution and targeted delivery. (Das et al., 2025) Finally, it is also noteworthy that repeated stimulus-response cycles may cause structural fatigue or performance degradation, reducing their long-term stability and reusability in complex pathological conditions. These limitations collectively impede the clinical advancement of these hydrogel systems.

## 3 Physical antibacterial hydrogels

In recent years, a distinctive class of physical antibacterial hydrogels has emerged as a prominent research focus, garnering substantial scientific attention. Although most systems still incorporate chemical compounds as adjuvants, they rely more heavily on non-chemical antimicrobial mechanisms and significantly mitigate the risk of bacterial resistance. Photo-responsive and sono-responsive hydrogels are particularly noteworthy. Unlike conventional stimulus-responsive hydrogels that serve as drug delivery platforms, these systems directly kill bacteria through physical mechanisms, hyperthermia from photothermal effects or mechanical disruption from ultrasonic waves, thus achieving efficient antimicrobial activity independent of pharmaceutical agents. When employed in conjunction with photosensitizers and sonosensitizers respectively, these hydrogels demonstrate remarkable synergistic bactericidal effects, offering a promising pathway for antibiotic alternatives.

### 3.1 Photo-responsive hydrogels

Driven by light energy, photo-responsive hydrogels function by sensing external light stimuli. Their antibacterial mechanism primarily relies on photothermal effects or photodynamic effects generated, respectively, by photothermal agents (PTAs) and photodynamic agents (PDAs, also called photosensitizers) incorporated within the matrix. (Jia et al., 2023) Common PTAs include gold nanoparticles, polydopamine, and graphene oxide derivatives (Li et al., 2022; Park et al., 2023), while typical PDAs include titanium dioxide, phthalocyanines, and porphyrin compounds. (Gnanasekar et al., 2023; Shing et al., 2021; Teng et al., 2025; Jiang et al., 2016; Xu et al., 2022; Cheng et al., 2023) Under near-infrared light irradiation, PTAs can produce localized hyperthermia via photothermal conversion to directly damage bacterial structures; PDAs can generate singlet oxygen (^1^O_2_) or other ROS molecules, accomplishing bacterial elimination through oxidative injury. (Jia et al., 2023; Gnanasekar et al., 2023; Pang et al., 2020) It bears noting that photothermally induced hyperthermia increases bacterial membrane permeability, thereby enhancing ROS penetration and creating synergistic “thermo-oxidative” effects. (Hao et al., 2022) Furthermore, the hydrophilic three-dimensional network structure of hydrogels provides a stable platform for collaboration between PTAs and PDAs (Jia et al., 2023). Pan et al. (2024) developed an intelligent hydrogel (HPCG/PFD) combining polydopamine-reduced graphene oxide (rGO-PDA) and glycine fullerene (Gly-C60) for diabetic foot ulcer treatment. This hydrogel could reach 54 °C within 5 minutes under near-infrared light and sustain ^1^O_2_ release for up to 2 hours post-irradiation, thus establishing a persistent antibacterial barrier and inhibiting biofilm formation.

The generation of ROS constitutes the crucial part of photodynamic therapy, yet excessive production would precipitate oxidative stress and tissue damage instead. Therefore, dynamic ROS scavenging has become critical for optimizing photo-responsive hydrogels. Building upon this, Ma et al. (2024) constructed an advanced MXene-based intelligent hydrogel incorporating polysalicylic acid components for dynamic ROS clearance. This system neutralizes free radicals and upregulates superoxide dismutase expression, restoring ROS levels to the physiological range within 2 hours post-phototherapy. *In vitro* validation showed the hydrogel maintained strong antibacterial activity while pronouncedly enhancing cell viability through activation of the antioxidant Nrf2 pathway. Animal studies further revealed that treated wounds exhibited orderly collagen fiber arrangement and markedly increased vascular density, achieving 97.5% healing in 12 days without scarring. This paradigm-shifting breakthrough addresses the current photodynamic therapy’s limitation of ROS “generation without elimination”.

In spite of their broad-spectrum antimicrobial activity and efficacy against drug-resistant pathogens, photo-responsive hydrogels remain constrained clinically. The restricted penetration depth of light sources confines these materials to superficial tissues and wound surfaces for anti-infective therapy. (Liu et al., 2018) Additionally, the limited coverage area renders them unsuitable for extensive infections. Moreover, though the high spatiotemporal resolution of light endows photo-responsive hydrogels with excellent controllability, enabling precise therapeutic modulation through wavelength and intensity adjustments, this advantage necessitates sophisticated optical equipment that may be prohibitively expensive and difficult to implement widely. Lastly, it is imperative to acknowledge that effective photothermal antibacterial treatment typically requires temperatures near 60 °C, which would potentially bring about patient discomfort and risk localized thermal tissue damage, (Hou et al., 2024) and the need for multiple treatment sessions also increases patient burden.

### 3.2 Sono-responsive hydrogels

Beyond photo-responsive hydrogels, sono-responsive hydrogels offer significant therapeutic potential resulting from their safety, cost-effectiveness, and non-invasive nature, (Zhou et al., 2022; Huang et al., 2024a) exhibiting superior antimicrobial efficacy when integrated with sonodynamic agents (SDAs, also called sonosensitizers). (Pang et al., 2020; Hou et al., 2023) The fundamental mechanism underlying pathogen elimination in these hydrogels centers on ultrasound-induced cavitation effects, where acoustic fields generate numerous microscopic bubbles within the liquid medium. These bubbles undergo cyclic expansion and violent collapse in response to periodic pressure oscillations induced by ultrasonic waves, thereby releasing intense energy that creates localized extreme temperatures (reaching thousands of Kelvin) and pressures (equivalent to hundreds of atmospheres), along with powerful shear forces and shock waves. (Garcia-Vargas et al., 2023; Guo et al., 2021; Li et al., 2025) This process delivers lethal damage to bacteria and can completely destroy resistant biofilm structures. (Hou et al., 2023; Kluge et al., 2022; Wang et al., 2020) Crucially, the spatial confinement and temporal transience of cavitation effects ensure that these phenomena operate exclusively at the microscopic scale to preclude damage to healthy tissues (Luan et al., 2024). Simultaneously, embedded SDAs are activated by ultrasound-triggered sonoluminescence or extreme temperatures. Similar to PDAs, activated SDAs produce substantial ROS molecules that attack bacterial targets (Pang et al., 2020). Additionally, high-frequency ultrasonic vibrations that disrupt bacterial membrane stability work synergistically with SDAs-generated ROS to maximize oxidative damage (Qi et al., 2025).

Unlike photo-responsive hydrogels, ultrasound demonstrates superior penetration through skin and deep tissues, positioning sono-responsive hydrogels as particularly valuable for treating deep infections (Pang et al., 2020). Ma et al. (2025) developed a sono-responsive platform utilizing manganese-doped carbon dots (MnCDs) as sonosensitizers. *In vitro* studies demonstrated 99.99% killing of MRSA in 8 mm-deep abscess models, with 3.8-fold better penetration than 808 nm near-infrared systems. Ex vivo porcine skin experiments confirmed ultrasonic penetration to a 3.5 cm depth, 8.7-fold greater than that of 808 nm laser systems, with cavitation effects enhancing SDAs accumulation in deep tissues.

Similarly, to address the dysregulation caused by excessive ROS generation in sonodynamic therapy, Wang et al. (2025b) invented ultrasound-responsive lithium-doped ZnO/PLLA piezoelectric microfibers (ZnLiPOI) with antioxidant 4-octyl itaconate (4OI) coating for dynamic “antibacterial-anti-inflammatory” therapy. Under intense ultrasound, the microfibers rapidly release ROS, eliminating 94.2% of *S. aureus* within 15 minutes. Subsequently, 4OI scavenges excess ROS and upregulates the KEAP1-Nrf2 pathway to reduce oxidative stress and accelerate inflammation resolution.

Last but not least, ultrasound can also serve as an effective trigger for targeted drug delivery, while ultrasonic thermal effects further enhance drug diffusion and release. Xiao et al. (2024) engineered a fibrin-based acoustically responsive composite hydrogel (ARS) using phase-change emulsions (W_1_/O/W_2_) where the inner phase (W_1_) carries drugs. Ultrasound vaporizes the perfluorohexane oil phase into microbubbles, disrupting the emulsion structure and releasing drugs. Zong et al. (2024) created an ultrasound-responsive hydrogel (XA@Ag/H) with a xanthan gum/sodium alginate dual-network framework loaded with Ag^+^ and self-assembled heparin-binding peptide nanoparticles. The Ca^2+^-mediated crosslinks enable ultrasound-triggered degradation into micrometer-scale fragments, allowing for deep drug penetration to disrupt bacterial biofilms and promote the healing of chronically infected diabetic wounds.

## 4 Biological antibacterial hydrogels

Traditional chemical and physical antibacterial methods indiscriminately kill both pathogenic bacteria and the normal skin microbiota, and thus biological antibacterial hydrogels have emerged as a groundbreaking approach that has captivated the scientific community. These hydrogels combat infections without the risk of flora imbalance by modulating the functions of host immune cells or incorporating specific living biological agents.

### 4.1 Immunomodulatory hydrogels

Macrophages, as a pivotal component of the innate immune system, are one of the principal effector cells driving sustained inflammatory infiltration in chronic wounds. (Maassen et al., 2023) Besides their formidable phagocytic capacity to recognize and clear pathogens and necrotic tissue, macrophages also play a central role in immune regulation. (Mass et al., 2023; Chen et al., 2023) Pro-inflammatory (M1) macrophages maintain localized inflammation through the secretion of inflammatory mediators including tumor necrosis factor-α and interleukin-1β, among others, whereas anti-inflammatory (M2) macrophages facilitate angiogenesis and tissue repair via the release of anti-inflammatory cytokines such as transforming growth factor-β and interleukin-10, among others. (Yang et al., 2023; Sun et al., 2021) Macrophage polarization refers to the dynamic process of phenotypic transition under different microenvironmental stimuli, endowing macrophages with remarkable functional plasticity. (Zhang et al., 2025b; Bianchi et al., 2025) In chronically infected wounds, the M1/M2 imbalance critically impairs wound healing (Qing, 2017; Chen et al., 2024), making the modulation of macrophage polarization essential for therapeutic intervention.

**Figure 7:**
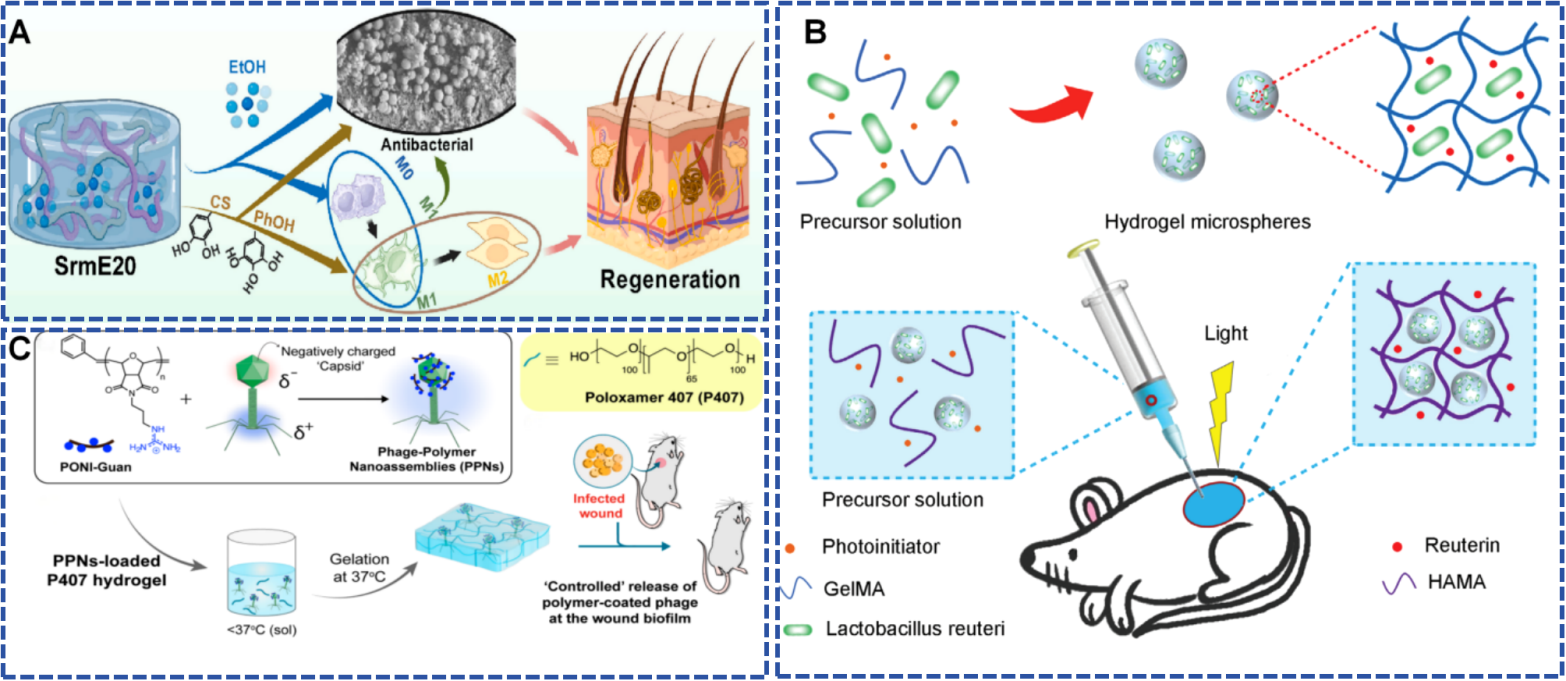
**(A)** Schematic illustration of the injectable immunoregulatory hydrogel that promotes the infected wound healing by sequentially regulating macrophage polarization (M0→M1→M2). Reproduced with permission. (Wang et al., 2024b) ^©^ 2024 The Authors. All rights reserved. Published by Elsevier B.V. on behalf of KeAi Communications Co. Ltd. under the Creative Commons CC BY-NC-ND 4.0 license. **(B)** The preparation of living probiotic hydrogels. Reproduced with permission. (Ming et al., 2021) ^©^ 2021 The Authors. All rights reserved. Published by Wiley-VCH GmbH under the Creative Commons CC BY license. **(C)** The synthesis of PPNs, the preparation of PPNs-loaded *P407* hydrogels, and their applications *in vivo* for treating MRSA-infected wound biofilms. Reproduced with permission. (Park et al., 2024) ^©^ 2024 American Chemical Society. All rights reserved.

Wang et al. (2024b) innovated an injectable immunomodulatory hydrogel (SrmE20) that promotes the healing of infected wounds by sequentially regulating macrophage polarization from M0 to M1 and subsequently to M2 phenotypes. The hydrogel combines anti-inflammatory components with pro-inflammatory solvents, enabling early antimicrobial activity through M1 macrophages and later tissue regeneration through M2 macrophages. *In vitro* studies showed that the system uses the volatility of ethanol to initially drive M1 polarization for rapid pathogen clearance. As ethanol depletes, phenolic hydroxyl groups and cationic species synergistically promote the M1-to-M2 transition, reducing inflammation and enhancing tissue repair (**Figure 7A**). This innovative strategy exploits hydrogel immunomodulatory properties to achieve a dynamic transition from antimicrobial to regenerative phases.

However, the traditional binary M1/M2 macrophage classification paradigm remains highly controversial. (Huang et al., 2022; Russell et al., 2019; Lee et al., 2018; Duan et al., 2025; Stewart et al., 2012; Hoffman et al., 2023; Starikova et al., 2023; Martynchyk et al., 2023) Macrophage polarization is critically dependent on dynamic changes within the wound microenvironment, with phenotypic regulation representing a complex and temporally evolving process. Accumulating evidence demonstrates that macrophages may traverse multiple intermediate states during this continuum (Stewart et al., 2012; Martynchyk et al., 2023; Lim et al., 2017; Chen et al., 2018; Guo et al., 2025; Chen et al., 2022; Zhang et al., 2025a), but their specific biological functions remain incompletely characterized, resulting in ambiguous delineation between M1 and M2 phenotypes. Additionally, key questions persist regarding the origins of M2 macrophages, the molecular mechanisms governing phenotypic polarization, and cellular subset proportions within heterogeneous macrophage populations. (Huang et al., 2022) These challenges constitute the primary bottleneck limiting the advancement of immunomodulatory hydrogels, rendering precise macrophage phenotype control for optimal outcomes extremely difficult. Consequently, an exhaustive elucidation of these multifaceted issues and the establishment of standardized classification criteria hold paramount importance for guiding the rational design of immunomodulatory hydrogels.

### 4.2 Probiotic-based hydrogels

The skin surface harbors both pathogenic and beneficial microbial communities, and probiotic bacteria create favorable microenvironments by secreting various metabolites and antimicrobial agents that promote their survival while inhibiting competing pathogens. (Harris-Tryon and Grice, 2022) Together with the normal skin tissue, these microorganisms form a complex microecosystem that serves as a vital barrier against external pathogenic invasion. (Harris-Tryon and Grice, 2022) However, conventional chemical and physical antibacterial treatments may disrupt the delicate microbial homeostasis of the skin and even trigger dysbiosis. (Ming et al., 2021; Xu et al., 2024) Live bacterial therapy has therefore engendered widespread investigative interest and has been successfully implemented in the diagnosis and treatment of wound infections. Given that hydrogels provide a stable environment conducive to nutrient transport and cellular proliferation, they are considered ideal carrier materials for viable microorganisms (Yu et al., 2022), thus giving rise to novel probiotic-based hydrogels.

The inherent bacterial characteristics of rapid colonization and proliferation pose significant challenges for the development of probiotic-based hydrogels, as poor microbial population control may trigger new ectopic infections. Additionally, the therapeutic efficacy of these systems may be undermined by the frequent clearance of exposed viable bacteria by the immune system. To address these issues, Ming et al. (2021) constructed an antibacterial hydrogel dressing loaded with viable probiotics that is designed to selectively inhibit pathogens while maintaining cutaneous microbial balance to accelerate the infected wound healing. The system employs emulsion polymerization to encapsulate *Lactobacillus reuteri* in methacrylated gelatin microspheres and then forms three-dimensional networks *in situ* through photoinitiated covalent crosslinking of methacrylated hyaluronic acid. This dual-barrier architecture protects probiotics from immune attack and prevents their migration to surrounding tissues while allowing the secretion of lactic acid and the antimicrobial agent *Reuterin* to suppress pathogenic bacteria such as *S. aureus* (**Figure 7B**). In an *S. aureus-infected* full-thickness wound model, the probiotic hydrogel achieved a 64% wound healing rate by day 4 and complete closure by day 10, versus 20% and 42% by day 4 in untreated and HA-only groups, respectively. Similarly, Xu et al. (2024) designed a highly active probiotic-based hydrogel containing encapsulated *Lactobacillus paracasei TYM202*, which inhibits pathogenic growth through lactic and acetic acid release while maintaining the *Firmicutes-Proteobacteria* balance to preserve skin microbiome stability.

Even with the significant achievements of probiotic-based hydrogels in infection control, microecological regulation, and the promotion of wound healing, major challenges still persist in their clinical application. The primary concern involves microbial equilibrium maintenance, especially in immunocompromised patients in whom probiotic dysregulation risks secondary infections. (Tom et al., 2022; Liu et al., 2024) Besides, interspecies competition among different bacterial strains may exacerbate microbial imbalances, further creating additional complications. These safety and controllability issues associated with live bacterial formulations have subjected these hydrogels to stringent ethical restrictions and regulatory oversight. From a translational perspective, when probiotic-based hydrogels are intended to prevent or treat skin infected wounds, they are generally asked to align with the regulatory concept of live biotherapeutic products, which implies drug level development requirements for clinical testing and consistent manufacturing, including well defined chemistry, manufacturing, and control specifications for strain identity, purity, potency, stability, and microbial contamination. (Cordaillat-Simmons et al., 2020; Charbonneau et al., 2020; Rouanet et al., 2020) Moreover, preserving probiotic viability and bioactivity within hydrogel matrices proves technically difficult, as bacterial strains may die or lose functionality over time, hampering the feasibility of long-term storage and transportation. Consequently, current research is actively addressing these safety and viability concerns by physically confining bacteria within microcarriers or dual barrier hydrogel architectures to reduce dissemination, applying whole genome sequencing and in silico screening to minimize risks related to virulence determinants or transferable antimicrobial resistance, and optimizing formulation and storage conditions to preserve viable counts and functional metabolite release over time. (Ming et al., 2021; Yu et al., 2022; Xu et al., 2024) Last but not least, there is insufficient fundamental research on probiotic mechanisms in wound environments and a lack of systematic analysis of the dynamic interactions between probiotics, host tissues, and pathogens, clarifying this issue is also crucial for advancing the development of probiotic-based hydrogels.

### 4.3 Bacteriophage-based hydrogels

Bacteriophages are obligate bacterial viruses that consist of a protein capsid enclosing single-stranded or double-stranded DNA or RNA genomes. (Havenga et al., 2022; Hitchcock et al., 2023) They present a compelling avenue for precision antibacterial intervention because of their exceptional host specificity, whereby they recognize bacterial surface-specific receptors to achieve precise targeting and ultimately lyse pathogenic bacteria. (Hitchcock et al., 2023; Zhang et al., 2022; Pertics et al., 2023; Bianchessi et al., 2024; Divya Ganeshan and Hosseinidoust, 2019) Bacteriophage therapy is particularly effective against drug-resistant infections owing to its rapid action and self-amplification within target bacteria. Only minimal doses are required, with infrequent replenishment, as phages replicate extensively upon bacterial invasion and naturally disappear after bacterial clearance. (Bianchessi et al., 2024; Divya Ganeshan and Hosseinidoust, 2019) In contrast to probiotics that may pose potentially uncontrolled risks, bacteriophages are generally considered safe for humans and help preserve the normal microbiota without causing dysbiosis. (Hitchcock et al., 2023; Bianchessi et al., 2024; Divya Ganeshan and Hosseinidoust, 2019) These advantages have made bacteriophage therapy an increasingly studied alternative for treating antibiotic-resistant bacterial infections.

Bacteriophage therapy has faced multiple challenges, including instability, poorly controlled release, and immune clearance, which make hydrogel encapsulation an appealing solution (Berkson et al., 2024; Briot et al., 2022; Pires et al., 2020). Conversely, bacterial biofilms greatly diminish phage recognition and lytic efficiency, (Pires et al., 2020) impairing the antibacterial effectiveness of phage-loaded hydrogels. Consequently, Park et al. (2024) engineered an antibacterial hydrogel based on phage-polymer nanoassemblies (PPNs) for managing drug-resistant biofilm infections. This platform exploited non-covalent assembly of cationic polyoxanorbornene polymer (PONI-Guan) with phage K to create positively charged nanocomposites that markedly enhanced phage penetration into MRSA biofilms. Notably, in a murine MRSA wound biofilm model, hydrogel-incorporated PPNs achieved a 1.5-log10 reduction in bacterial load, compared with a 0.5-log10 reduction using phage K in the same hydrogel matrix. Mechanistically, the cationic polymer augmented phage penetration through electrostatic interactions with negatively charged biofilm matrix components, with confocal microscopy confirming widespread phage distribution throughout the entire biofilm architecture. Furthermore, PPNs were incorporated into thermosensitive *Poloxamer 407* hydrogels for sustained phage release and prolonged local antibacterial action (**Figure 7C**). This work represents a breakthrough integration of nanocarrier engineering with hydrogel delivery to resolve the core bottlenecks of biofilm penetration and local retention in phage therapy.

Phage therapy was once regarded as one of the most potential solutions to the global challenge of “*superbugs*”, yet its efficacy is now increasingly jeopardized by bacterial anti-phage mechanisms such as CRISPR-Cas and restriction-modification systems. (Pires et al., 2020; Georjon and Bernheim, 2023; Yuan et al., 2023) Bacteria possess their own defense systems against phage invasion, and the target specificity that constitutes an advantage of phage therapy can paradoxically limit therapeutic outcomes. (Hitchcock et al., 2023; Divya Ganeshan and Hosseinidoust, 2019; Pires et al., 2020; Yuan et al., 2023) Overcoming this requires future efforts directed toward engineering modified or synthetic phage variants, or implementing multivalent phage cocktails to circumvent bacterial resistance (Pires et al., 2020; Yuan et al., 2023; Strathdee et al., 2023; Hitchcock et al., 2023; Divya Ganeshan and Hosseinidoust, 2019). Additionally, since therapeutic phages are self-replicating biological entities, their pharmacokinetic and pharmacodynamic profiles are significantly more complex than those of conventional antibiotics, and clarifying these parameters is crucial for translating bacteriophage-based hydrogels into clinical applications (Nang et al., 2023).

## 5 Summary and perspectives

This review systematically examines recent advances in antibacterial hydrogels for treating skin-infected wounds, categorizing them into three principal antimicrobial strategies based on their mechanisms of action: chemical, physical, and biological approaches. Chemical antibacterial hydrogels incorporate natural or synthetic antimicrobial agents in conjunction with stimuli-responsive technologies to achieve sustained and broad-spectrum bactericidal activity. Physical antibacterial hydrogels leverage non-pharmacological modalities, such as photothermal or ultrasonic techniques, to directly disrupt pathogen and biofilm structures, thereby effectively mitigating the development of antimicrobial resistance. Biological antibacterial hydrogels that are centered on immunomodulatory functions or living biological agents combat infection by modulating host immune responses or reconstituting the wound microenvironment. There is a concise quantitative snapshot compiling representative preclinical antibacterial and wound-healing endpoints reported for the different hydrogel strategies discussed in this review to demonstrate their efficacy in preclinical settings (**Table 2**). While each strategy delivers distinct advantages, they still encounter multifaceted obstacles, including long-term stability, biosafety concerns, response precision, cost-effectiveness, and limited clinical translation. It is noteworthy that most current research remains confined to the laboratory, though certain materials (such as silver ion-based, chitosan-based, and silk fibroin hydrogels) have shown promising clinical potential. Data on novel antibacterial hydrogels for skin-infected wounds are derived predominantly from animal models, with large-scale human clinical trials remaining scarce. Furthermore, most studies assess only overall antimicrobial efficacy and wound healing, neglecting detailed analyses of efficacy-time relationships.

**Table 2:** Selected preclinical examples cited in this review provide representative quantitative efficacy benchmarks of different antibacterial hydrogel strategies.

Strategies	Representative examples	Quantitative antibacterial or wound-healing endpoints (as reported)
Chemical Antibacterial Hydrogels	Jelleine-1 self-assembling antimicrobial peptide (AMP) hydrogel (Zhou et al., 2023)	>98.5% killing efficacy against methicillin-resistant Staphylococcus aureus (MRSA), E. coli, and C. albicans *in vitro*.
	Ag-G4/hemin DNAzyme-modified Ag nanocluster hydrogel (Zhu et al., 2023)	Eliminated 99.9% of MRSA/S. aureus/E. coli; achieved a five-log bacterial reduction within 24 h in MRSA-infected wounds.
	CPO-Alg hydrogel (Huang et al., 2024b)	>99.99% bactericidal efficacy against E. coli and S. aureus at early stage; 100% wound closure within 14 days; +66.7% collagen deposition versus controls.
Physical Antibacterial Hydrogels	HPCG/PFD photo-responsive hydrogel (Pan et al., 2024)	Reached 54 °C within 5 min under near-infrared (NIR); sustained singlet oxygen (^1^O_2_) release for up to 2 h post-irradiation to inhibit biofilm formation.
	MXene-based photo-responsive hydrogel with dynamic reactive oxygen species (ROS) clearance (Ma et al., 2024)	Achieved 97.5% wound healing within 12 days without scarring in animal studies.
	Ultrasound-responsive ZnLiPOI piezoelectric microfibers hydrogel system (Wang et al., 2025b)	Eliminated 94.2% of S. aureus within 15 min under ultrasound stimulation.
Biological Antibacterial Hydrogels	Living probiotic hydrogel (LRHA) encapsulating Lactobacillus reuteri (Ming et al., 2021)	In an S. aureus-infected full-thickness wound model, the probiotic hydrogel achieved a 64% wound healing rate by day 4 and complete closure by day 10, versus 20% and 42% by day 4 in untreated and HA-only groups, respectively.
	Phage-polymer nanoassemblies (PPNs) in Poloxamer 407 hydrogel (Park et al., 2024)	*In vitro*, ~3-log10 bacterial reduction against MRSA biofilms; *in vivo* murine MRSA wound biofilm model, 1.5-log10 reduction in bacterial load versus 0.5-log10 with phage-only hydrogel.

Endpoints are reported from individual studies and are not derived from direct head-to-head comparisons; variations in pathogens, models, dosing, and outcome definitions may limit cross-study comparability.

AMP, antimicrobial peptide; MRSA, methicillin-resistant Staphylococcus aureus; NIR, near-infrared; 1O2, singlet oxygen; ROS, reactive oxygen species.

Future antibacterial hydrogels necessitate a research paradigm grounded in multifunctional optimization, technological integration, and interdisciplinary collaboration to advance personalized precision medicine. To avoid antibiotic-induced resistance, combinatorial therapies will be essential for managing multidrug-resistant infections. Equally important is maximizing the synergistic interplay between antimicrobial efficacy and tissue regeneration. For instance, integrating antibacterial, anti-inflammatory, and pro-angiogenic properties may address the complex pathology of chronic wounds. Concurrently, engineering dynamically responsive controlled-release systems with heightened sensitivity to pathological microenvironments will substantially improve drug bioavailability and treatment kinetics.

More strikingly, emerging technologies are driving innovation in antibacterial hydrogels. Three-dimensional bioprinting enables the precise fabrication of wound-matched dressings tailored to specific geometries to facilitate customized interventions. (Fang et al., 2023) Chemical modification and nanocarrier encapsulation via nanotechnology not only effectively enhance biocompatibility and long-term stability, but also improve drug-loading efficiency and tissue penetration, thus amplifying both the depth and precision of localized treatment. (Amiri et al., 2021) Smart hydrogels incorporating biosensors and feedback systems hold considerable promise for establishing therapeutic platforms integrated with real-time monitoring and responsive drug release that are particularly suitable for chronic infected wound management. Simultaneously, precise drug-controlled release can also avoid the toxic side effects of the medication. (Ge et al., 2023; Lei et al., 2024) Furthermore, interdisciplinary collaboration spanning materials science, biomedicine, and information technology will provide robust support for the development of multifunctional, intelligent, and clinically translatable next-generation hydrogels. Finally, expediting large-scale, multicenter randomized controlled clinical trials for safety validation, alongside streamlining ethical reviews and establishing comprehensive regulatory frameworks with standardized evaluation systems, will promote the clinical translation of these materials from bench to bedside, with priority given to advancing injectable, biodegradable, and sustained-release antibacterial hydrogels into preclinical studies. As a frontier antibiotic-alternative strategy, antibacterial hydrogels are anticipated to provide more efficacious, safer, and more personalized solutions for infected wound management.
